# Electrospun Nanofiber Meshes With Endometrial MSCs Modulate Foreign Body Response by Increased Angiogenesis, Matrix Synthesis, and Anti-Inflammatory Gene Expression in Mice: Implication in Pelvic Floor

**DOI:** 10.3389/fphar.2020.00353

**Published:** 2020-03-24

**Authors:** Shayanti Mukherjee, Saeedeh Darzi, Kallyanashis Paul, Fiona L. Cousins, Jerome A. Werkmeister, Caroline E. Gargett

**Affiliations:** ^1^The Ritchie Centre, Hudson Institute of Medical Research, Clayton, VIC, Australia; ^2^Department of Obstetrics and Gynaecology, Monash University, Clayton, VIC, Australia

**Keywords:** mesenchymal stem cells, pelvic organ prolapse, electrospinning, nanofiber mesh, tissue engineering, foreign body response, gene expression, foreign body giant cells

## Abstract

**Purpose:**

Transvaginal meshes for the treatment of Pelvic Organ Prolapse (POP) have been associated with severe adverse events and have been banned for clinical use in many countries. We recently reported the design of degradable poly L-lactic acid-co-poly ε-caprolactone nanofibrous mesh (P nanomesh) bioengineered with endometrial mesenchymal stem/stromal cells (eMSC) for POP repair. We showed that such bioengineered meshes had high tissue integration as well as immunomodulatory effects *in vivo*. This study aimed to determine the key molecular players enabling eMSC-based foreign body response modulation.

**Methods:**

SUSD2^+^ eMSC were purified from single cell suspensions obtained from endometrial biopsies from cycling women by magnetic bead sorting. Electrospun P nanomeshes with and without eMSC were implanted in a NSG mouse skin wound repair model for 1 and 6 weeks. Quantitative PCR was used to assess the expression of extracellular matrix (ECM), cell adhesion, angiogenesis and inflammation genes as log_2_ fold changes compared to sham controls. Histology and immunostaining were used to visualize the ECM, blood vessels, and multinucleated foreign body giant cells around implants.

**Results:**

Bioengineered P nanomesh/eMSC constructs explanted after 6 weeks showed significant increase in 35 genes associated with ECM, ECM regulation, cell adhesion angiogenesis, and immune response in comparison to P nanomesh alone. In the absence of eMSC, acute inflammatory genes were significantly elevated at 1 week. However, in the presence of eMSC, there was an increased expression of anti-inflammatory genes including *Mrc1* and *Arg1* by 6 weeks. There was formation of multinucleated foreign body giant cells around both implants at 6 weeks that expressed CD206, a M2 macrophage marker.

**Conclusion:**

This study reveals that eMSC modulate the foreign body response to degradable P nanomeshes *in vivo* by altering the expression profile of mouse genes. eMSC reduce acute inflammatory and increase ECM synthesis, angiogenesis and anti-inflammatory gene expression at 6 weeks while forming newly synthesized collagen within the nanomeshes and neo-vasculature in close proximity. From a tissue engineering perspective, this is a hallmark of a highly successful implant, suggesting significant potential as alternative surgical constructs for the treatment of POP.

## Introduction

Pelvic Organ Prolapse (POP) is a debilitating urogynecological pelvic floor disorder that significantly impacts the quality of lives of 50% of parous women aged over 50 years (Nygaard et al., 2008). POP is mainly results from vaginal birth injury (Urbankova et al., 2019), which over time leads to herniation of pelvic organs, such as uterus, bladder, and bowel into the vagina. Symptoms include difficulty in passing urine and bowel motions, sexual dysfunction, feeling of a vaginal bulge, and urinary and bowel incontinence (Iglesia and Smithling, 2017). While first line conservative management using pelvic floor exercises and pessaries may delay disease progression (Li et al., 2016), it does not eliminate the need for surgical intervention for many women. Until recently, non-degradable polypropylene (PP) vaginal meshes were commonly used for reconstructive surgery to mitigate native tissue repair failures (Milani et al., 2018). However, regulatory authority warnings and recent reports indicate high adverse event rates and risks of complications such as mesh erosion and exposure (Mironska et al., 2019). A growing body of evidence shows implant failures and have established that prolonged inflammation and undesirable foreign body response (FBR) are associated with complications in patients (Deprest et al., 2009; Claerhout et al., 2010; Brown et al., 2015; Jallah et al., 2016; Nolfi et al., 2016; Tennyson et al., 2019). Such FBRs and associated adverse effects of transvaginal meshes were deemed to out-weigh PP benefits and therefore led to the ban on transvaginal meshes in Australia, UK, and USA by regulatory authorities, with no alternative treatments on the horizon. At present, there are no optimal therapies for POP. Therefore, more reliable treatment measures that promote tissue healing and repair without piquing deleterious FBR are pivotal for the treatment of POP (Siddiqui et al., 2018).

In nature, *in vivo* cell behavior and vaginal tissues are supported by the micro/nanoscale architecture of the ECM (Sridharan et al., 2012) that provides a larger surface area to adsorb proteins and more binding sites for cell membrane receptors and adhesion molecules. The standard clinical PP mesh biomaterial lacks a biomimetic character. They disrupt the vaginal microenvironment (Liang et al., 2013; Liang et al., 2015; Jallah et al., 2016) rather than mimicking its nanoarchitecture, evoking undesired complications. To overcome mesh erosion, vaginal implants must promote rather than impede cell-matrix interactions. The primary cause of complications resulting from PP mesh implants have been attributed to the prolonged chronic inflammation and poor tissue integration associated with mechanically inferior non-degradable implants (Nolfi et al., 2016; Tennyson et al., 2019). The tissue microenvironment comprises structural and functional components (e.g. collagens and elastin) that provide a scaffold to hold cells together through numerous chemical and physical stimuli at the molecular level. Nanofabrication of scaffolds recapitulates such biomimetic nanoscale architectural cues (Mukherjee et al., 2013; Liu et al., 2018a). As a result, meshes designed with nanoscale fibers using electrospinning techniques promote cell-cell and cell-biomaterial interactions. Given that current PP meshes bear little structural or biological resemblance to native vaginal tissue, we and others have shown that nanostructured meshes that impart biomimetic properties can improve mesh integration, overcome erosion and hold significant promise in POP reconstructive surgery (Sartoneva et al., 2012; Wu et al., 2016; Vashaghian et al., 2017; Gargett et al., 2019; Mangir et al., 2019; Mukherjee et al., 2019b).

Irrespective of the composition and fabrication technique, biomaterials elicit an FBR after implantation in the body (Mukherjee et al., 2019a; Hympanova et al., 2020). This response is a cascade of dynamic cellular processes involving several genes influencing the milieu of the tissue microenvironment that ultimately determines the fate of the implant and healing process. Mesenchymal stem/stromal cells (MSCs) are clonogenic, multipotent cells, widely recognised for their ability to promote tissue repair and regeneration (Dimarino et al., 2013; Ulrich et al., 2013; Le Blanc and Davies, 2015; Gargett et al., 2016). Therefore, cell based therapies for pelvic floor tissue repair, although less explored, hold significant potential for POP treatment (Darzi et al., 2016b; Emmerson and Gargett, 2016; Gargett et al., 2016; Callewaert et al., 2017; Gargett et al., 2019). Nonetheless, while undifferentiated MSCs mitigate inflammation and influence reparative processes (Kode et al., 2009; Le Blanc and Davies, 2015), several clinical trial outcomes have highlighted that mere injection of such therapeutic cells into damaged tissue leads to a rapid loss of MSC, preventing optimal repair (Dimmeler et al., 2014; Sharma et al., 2014). Bioengineering using biomimetic degradable nanofiber meshes that mimic natural ECM to allow entrapment and persistence of seeded MSCs will likely yield superior vaginal constructs with a controlled and anti-inflammatory immune response (Gargett et al., 2019).

We discovered perivascular MSCs in the endometrial lining of the uterus (eMSC) and identified a unique marker, SUSD2, to isolate these rare perivascular cells (Gargett et al., 2016). We also discovered that a small molecule, A83-01, maintains eMSC' undifferentiated state during culture expansion, required for clinical use (Gurung et al., 2015; Gurung et al., 2018). We have established that eMSC have reparative capacity, reduce fibrosis and the FBR to nondegradable polyamide mesh by influencing macrophage polarization switching from an M1 to M2 phenotype in rodent and ovine models (Ulrich et al., 2014; Darzi et al., 2018; Emmerson et al., 2019). More recently, we have also shown that eMSC improve the tissue integration, cellular infiltration and overall FBR response to degradable nano/microstructured meshes (Mukherjee et al., 2019b; Paul et al., 2019). The beneficial effects of eMSC are characterized by upregulation of M2 markers such as CD206 and *Arg1*, *Mrc1*, and *Il10* genes in tissue macrophages, as well as reducing their secretion of inflammatory cytokines Il-1β and Tnf-α (Darzi et al., 2018). However, the key players in mediating eMSC paracrine effects on cellular migration and recruitment remain largely unknown. Furthermore, how eMSC mediate M2 immunomodulatory responses during the FBR after implantation of bioengineered constructs also remains unknown. In general, the FBR to tissue engineered constructs are often limited to measuring the *in vivo* capsule thickness and is poorly understood.

Recently, we reported the design of novel nanostructured degradable poly L-lactic acid-co-poly ε-caprolactone or PLCL meshes (P nanomesh) tissue engineered with reparative mesenchymal stem/stromal cells from endometrium (Mukherjee et al., 2019b). In this study, we assess the potential of these newly designed degradable nanofiber meshes tissue engineered with these therapeutic cells to influence macrophage mediated FBR and promote key reparative processes such as angiogenesis, cellular adhesion, extracellular matrix (ECM) synthesis as well as its regulation using gene expression profiling and histology in a subcutaneous mouse model. From a clinical perspective, it is not only important to design novel constructs for POP treatment, but also critical to understand their FBR pattern and tissue repair process that likely varies with different components and their degree of involvement (Mukherjee et al., 2019a). Such detailed understanding is also pivotal to the long term efficacy of all medical devices and the lack of this knowledge may potentially disrupt clinical practices as exemplified by the rise and fall of pelvic PP mesh usage (Heneghan et al., 2017). In this study we provide an in-depth assessment of changes in gene expression associated with eMSC-nanobiomaterial therapy. In particular, we have quantified the *in vivo* gene expression associated with ECM formation and regulation, cell adhesion, angiogenesis and the FBR to PLACL Nanomesh (P Nanomesh) with and without eMSC. We have also shown the histological effects arising from the gene expression profile of eMSC based surgical constructs, including angiogenesis and ECM formation. To our knowledge, this is the first study to show such detailed impact of eMSC based tissue engineered degradable nanostructured scaffolds *in vivo*.

## Methods

### Ethics

All SUSD2^+^ eMSC were isolated from endometrial biopsies obtained from seven women undergoing laparoscopic surgery for nonendometrial gynecological conditions and had not taken hormonal treatment for three months before surgery. Samples were collected following written informed consent as per approval from the Monash Health and Monash University Human Research Ethics committees (09270B). All methods were performed in accordance with National Health and Medical Research Council guidelines. Each patient biopsy was used to generate a single eMSC cell line and served as n=1.

### Fabrication of Nanomeshes

Nanofiber meshes of PLACL were fabricated by electrospinning as described in our previous report (Mukherjee et al., 2019b). PLACL polymer (Resomer, Evonik) was dissolved in 1,1,1,3,3,3-hexafluoro-2-propanol (HFIP) on a magnetic stirrer to form 10% (w/v) clear solution. Syringe (Terumo Corporation, Japan) with this solution was attached to 23 G blunted stainless-steel needle (Terumo Corporation, Japan) for electrospinning, using a syringe pump (NE1000, New Era Pump Systems, Inc. USA) at a controlled flow rate of 1 ml/h and voltage of 18 kV (DC voltage power supply, Spellman SL150, USA) to collect nanofibers at a distance of 12.5 cm from the needle tip to the collector. The fibers were collected on grounded aluminum foil and dried for at least 1 week in a vacuum oven prior to experimental use.

### Scanning Electron Microscopy

Samples of only nanofiber meshes were directly sputter coated with a thin platinum sputter coating layer (Cressington 208 HR, UK) for 120 s. All specimens were examined under the scanning electron microscope (Nova NanoSEM, FEI, USA) at an accelerating voltage of 10 kV and images were quantified by Image J software.

### Atomic Force Microscopy

Atomic force microscopy (AFM) using a FastScan AFM (Bruker, Billerica, MA, USA) in PeakForce tapping mode and FastScan C probes with a nominal 5 nm tip radius and spring constant of 0.8 N/m. For imaging, 512 × 512 pixel resolution and a 2-Hz scan rate was used to measure n=3 samples and pointed AFM tip at five ROIs (region of interest) of 5 μm^2^ area. Images were processed using Nanoscope Analysis software.

### Isolation, Expansion, and Labelling of eMSC

Endometrial tissue was obtained from seven healthy women (no endometrial pathology) who had not used hormones for minimum three months. SUSD2^+^ eMSC were isolated according to our established protocols (Darzi et al., 2018; Mukherjee et al., 2019b). Briefly, endometrial tissue underwent enzymatic digestion using Collagenase I and DNAs I (Worthington-Biochemical Corporation) for 1 h at 37°C. The stromal fraction and red blood cells were separated from epithelial fraction using a 40 μM sieve (BD Bioscience-Durham) and Ficoll paque (GE Healthcare Bioscience-Bio-Sciences AB) gradient, respectively. The isolated stromal cells were incubated with PE anti human SUSD2 antibody (Biolegend) for 30 min at 4°C followed by incubation with anti-PE labelled magnetic beads (Miltenyi Biotec) for 20 min. PE labelled SUSD2^+^ eMSC were sorted using a column and magnet (Miltenyi Biotec). SUSD2^+^ eMSC were cultured and expanded in 10% FBS DMEM F12 (Invitrogen) supplied with growth factor bFGF (Peprotech) for 2–4 passages. Before *in vivo* implantation, eMSC were permanently labelled with mCherry lentivirus vector according to our published protocols (Darzi et al., 2018; Mukherjee et al., 2019b). Briefly, Lentivirus was generated using three plasmids; pLVX-IRES-mCherry (lentivirus plasmid which contains mCherry gene) (clontech-6312237), packaging plasmids; pSPAX2 that encodes capsid (Addgene 12260) and pMD2.G that encodes reverse transcriptase for lentivirus replication (Addgene 12259), into 293T cells. Transfection was performed using TransIT-X2 (Mirus) transfection reagent according to manufacturer's protocols.

### Animal Surgery and Tissue Collection

The experimental procedure and mouse husbandry was approved by Monash Medical Centre Animal Ethics Committee A (2017/05). NSG mice were housed in the animal house at Monash Medical Centre according to the National Health and Medical Research Council of Australia guidelines for the care and use of laboratory animals and were provided sterile food and water under controlled environmental conditions. NSG mice were divided in two experimental groups P and P+eMSC and two time-points; 1 and 6 weeks (seven mice/group). The mice were anaesthetized using 3% w/v Isoflurane^®^ and carprofen (5 mg/kg body weight) was used as analgesia. The abdomen was shaved and disinfected with 70% ethanol. A longitudinal 1.2 cm skin incision was performed in the lower abdomen and the skin was separated from the fascia by blunt dissection to make two pockets on each side of the midline. The P nanomesh was implanted into two pockets of each animal, and mCherry labelled eMSC was added on top of the nanomesh using a 50µl pipette tip. Meshes were sutured to the abdominal fascial layer using 6–0^®^ monofilament sutures (Ethicon) on two ends and. The skin was closed with 6–0^®^ monofilament sutures (Vicryl). Following 1 or 6 weeks the animals were euthanized in a CO_2_ chamber and tissues were collected for analysis. Some animals were reused from our previous study (Mukherjee et al., 2019b) to comply with Monash Medical Centre Animal Ethics Committee's reuse and reduce usage policy. However, the tissue portions of the animals used for analysis have not been used in any other study.

### Histology

Tissue sections containing constructs were fixed in 10% formalin and processed by the Monash Histology Platform at the Monash Health Translation Precinct (MHTP). Formalin-fixed tissues were processed to paraffin, sectioned (5 µm) and placed on super frost slides. Histological H&E, Picrosirius red staining and immunohistochemistry was performed by Monash Histology Platform at MHTP. For H&E staining, slides were dewaxed using xylene and stained in Haemtoxylin for 7 min. After washing in tap water, they were blued in ammoniated water for 30 seconds and stained in alcoholic Eosin for 7 min. For Picro Sirius red staining, slides were dewaxed using xylene and fixed in Bouin's fixative for 1 h. After washing in tap water, they were stained with Picro Sirius red for 1 h at room temperature followed by washing and mounting. For immunohistochemistry staining, slides were dewaxed and underwent citrate buffer antigen retrieval for 30 min followed by endogenous peroxidase blocking step using 0.3% v/v H_2_O_2_. Slides were then incubated with protein blocker (Dako, USA) for 40 min and washed with 1× PBS. Primary antibodies were diluted and incubated for 1 h at room temperature. HRP labelled anti mouse was used as secondary antibody for 30 min and the nucleus stained with Hematoxylin. Details of antibodies are listed in **Table1**. The sections are scanned using Aperio Digital pathology scanner (Leica) at 40X and analyzed using Imagescope software to identify the stains.

**Table 1:** Details of antibodies used in immunohistochemistry.

Primary Antibody	Isotype	Supplier	Dilution
CD206	Rabbit polyclonal	Abcam	1/2500
mCherry	Rat IgG1	Life technology	1/100

### qPCR Fluidigm Biomark

Animal tissues were collected in RNA*later* (ThermoFisher) and stored in 4°C for 24 h followed by storage in −80°C. Samples were weighed and total RNA was extracted using RNeasy mini kit (Qiagen) as per manufacturer's protocol to prepare cDNA. Prior to fluidigm qPCR, preamplification was used to increase the number of copies of each gene to detectable levels as detailed in Fluidigm Gene Expression. Taqman assays were firstly pooled by combining 2 μl of each of the 94 20X Taqman assays and 12 μl Tris EDTA buffer pH 8.0 for a final volume of 200 μl. The final concentration of each assay was 0.2X (180nM). 3.75 μl of Sample Premix (Life Technologies TaqMan^®^ PreAmp Master Mix and Pooled Taqman assays) was combined with 1.25 μl of each of the 87 cDNA samples and 8 RT negative samples for a final reaction volume of 5 μl per sample. A no template control from Single Cell Genomics Centre was also included and all 96 samples were preamplified for 14 cycles. Following preamplification, reaction products were diluted 1:5 by adding 20 μl Tris EDTA buffer pH 8.0 to the final 5 μl reaction volume for a total volume of 25 μl. Assays and Samples were combined in a 96.96 Dynamic array IFC according to Fluidigm^®^ 96.96 Real-Time PCR Workflow. Five microliter of each assay at a final concentration of 10× was added to each assay inlet port and 5 μl of diluted sample to each sample inlet port according to the Chip Pipetting Map (GE 96×96 Standard v2). Data were analyzed using with Fluidigm Real-Time PCR analysis software (V4.1.1) to obtain Ct values. Primers are detailed in **Table 2**. Target gene expression was normalized to 18sRNA and relative gene expression and fold change was calculated using the 2^-ΔΔCT^ method.

**Table 2:** Details of qPCR Primers (mouse genes).

Gene name	TaqMan Code
*Ang1*	Mm00456503_m1
*Ang2*	Mm00545822_m1
*Fgf1*	Mm00438906_m1
*Fgf2*	Mm00433287_m1
*Fgfr3*	Mm00433294_m1
*Ctgf*	Mm01192933_g1
*Mmp2*	Mm00439498_m1
*Mmp9*	Mm00442991_m1
*Mmp19*	Mm00491296_m1
*Pdgfa*	Mm01205760_m1
*Timp1*	Mm00441818_m1
*Timp2*	Mm00441825_m1
*Timp3*	Mm00441826_m1
Timp4	Mm01184417_m1
*Tgfa*	Mm00446232_m1
*Tgfb1*	Mm00441724_m1
*Tgfb2*	Mm00436955_m1
*Tgfb3*	Mm00436960_m1
*Tgfbr1*	Mm00436964_m1
*Vegfa*	Mm00437304_m1
*Serpine1*	Mm00435860_m1
*Itgb1*	Mm01253230_m1
*Itgb2*	Mm00434513_m1
*Ccl1*	Mm01545656_m1
*Ccl2*	Mm00441242_m1
*Ccl3*	Mm00441258_m1
*Ccl4*	Mm00443111_m1
*Ccl5*	Mm01302428_m1
*Ccl7*	Mm00443113_m1
*Ccl11*	Mm00441238_m1
*Ccl12*	Mm01617100_m1
*Ccl17*	Mm01244826_g1
*Ccl19*	Mm00839967_g1
*Cxcl1*	Mm04207460_m1
*Cxcl2*	Mm00436450_m1
*Cxcl5*	Mm00436451_g1
*Cxcl9*	Mm00434946_m1
*Cxcl10*	Mm00445235_m1
*Cxcl11*	Mm00444662_m1
*Cxcl12*	Mm00445553_m1
*Ccr1*	Mm00438260_s1
*Ccr2*	Mm99999051_gH
*Ccr3*	Mm01216172_m1
*Ccr5*	Mm01963251_s1
*Ccr7*	Mm00432608_m1
*Cxcr2*	Mm99999117_s1
*Cxcr3*	Mm00438259_m1
*Il1a*	Mm00439620_m1
*Il1b*	Mm00434228_m1
*Il4ra*	Mm01275139_m1
*Il6*	Mm00446190_m1
*Tnf*	Mm00443258_m1
*Il10*	Mm00439616_m1
*Nos1*	Mm01208059_m1
*Nos2*	Mm00440485_m1
*Cd86*	Mm00444540_m1
*CD80*	Mm01344159_m1
*Arg1*	Mm00475988_m1
*Mrc1*	Mm00485148_m1
*Tnfa*	Mm99999068_m1
*Cd44*	Mm01277163_m1
*Cdh1*	Mm01247357_m1
*Cdh2*	Mm01162497_m1
*Cd49B/Itga2*	Mm00434371_m1
*Icam*	Mm00516023_m1
*Vcam1*	Mm01320970_m1
*Col6a1*	Mm00487160_m1
*Col6a2*	Mm00521578_m1
*Col6a3*	Mm00711678_m1
*Col6a6*	Mm00556810_m1
*Col1a1*	Mm00801666_g1
*Col3a1*	Mm01254476_m1
*Rn18s*	Mm03928990_g1
*Gapdh*	Mm03302249_g1

### Data Analysis and Statistics

Fold Change in gene expression was calculated in comparison to sham controls. Statistical analysis was performed using GraphPad Prism v8. Data were analyzed using non-parametric Mann-Whitney U test (comparison between P and P+eMSC). Data are presented as median and value of P ≤ 0.0513 was considered to be statistically significant.

## Results

### Fabrication and Characterization of Nanomesh

**Figure 1:**
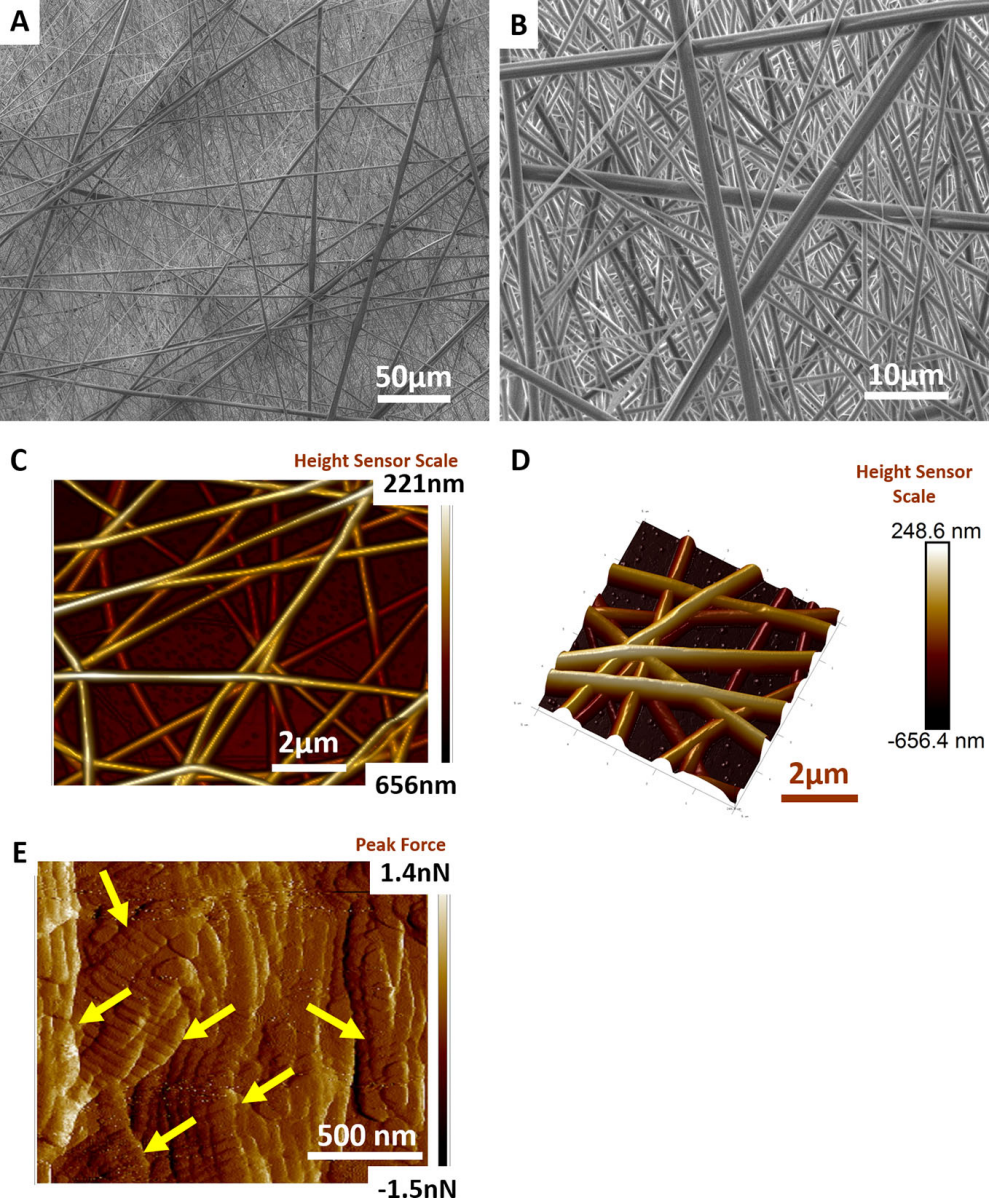
Electrospun Nanofiber mesh. Scanning electron micrographs of PLCL nanofiber mesh structure at **(A)** 1000× magnification **(B)** 5000× magnification. Atomic force micrograph reveals structure of **(C)** randomly laid nanofiber meshes at two-dimensional (2D) view and **(D)** 3D view **(E)** vaginal extracellular matrix (ECM) structure revealing arrangement of collagen fibrils (yellow arrows).

Degradable nanostructured meshes were fabricated from poly (L-lactic acid)-co-poly(ε-caprolactone) (P Nanomesh), given their acceptance in medical devices, using electrospinning to mimic the precise features of native tissue dimensions as per our previous studies (Mukherjee et al., 2011; Mukherjee et al., 2019b). Electrospinning enabled the design of nanofibers of PLCL (**Figure 1**) that produced a mesh which macroscopically appeared like thin facial tissue paper. Scanning electron microscopy (SEM) micrographs confirmed that P nanomeshes had an ultrafine and beadless morphology (**Figure 1A**) with an average fiber diameter of 585 nm as previously reported (Mukherjee et al., 2019b). The nanomeshes were highly porous (**Figure 1B**) and had three-dimensional structure of randomly layered fibers to form sheets of ~406 nm in thickness (**Figures 1C, D**). The fabricated P nanomesh structures closely resembled the human vaginal microstructure at the nanoscale, comprised of collagen fibril structures (**Figure 1E** and **Figure S1**) that ranged from 55–130 nm depending on the patient age and POP severity, and are arranged in bundles 2–3 µm thick (Kim et al., 2016).

### eMSC Increase Synthesis of New ECM Within Nanomeshes *In Vivo*

**Figure 2:**
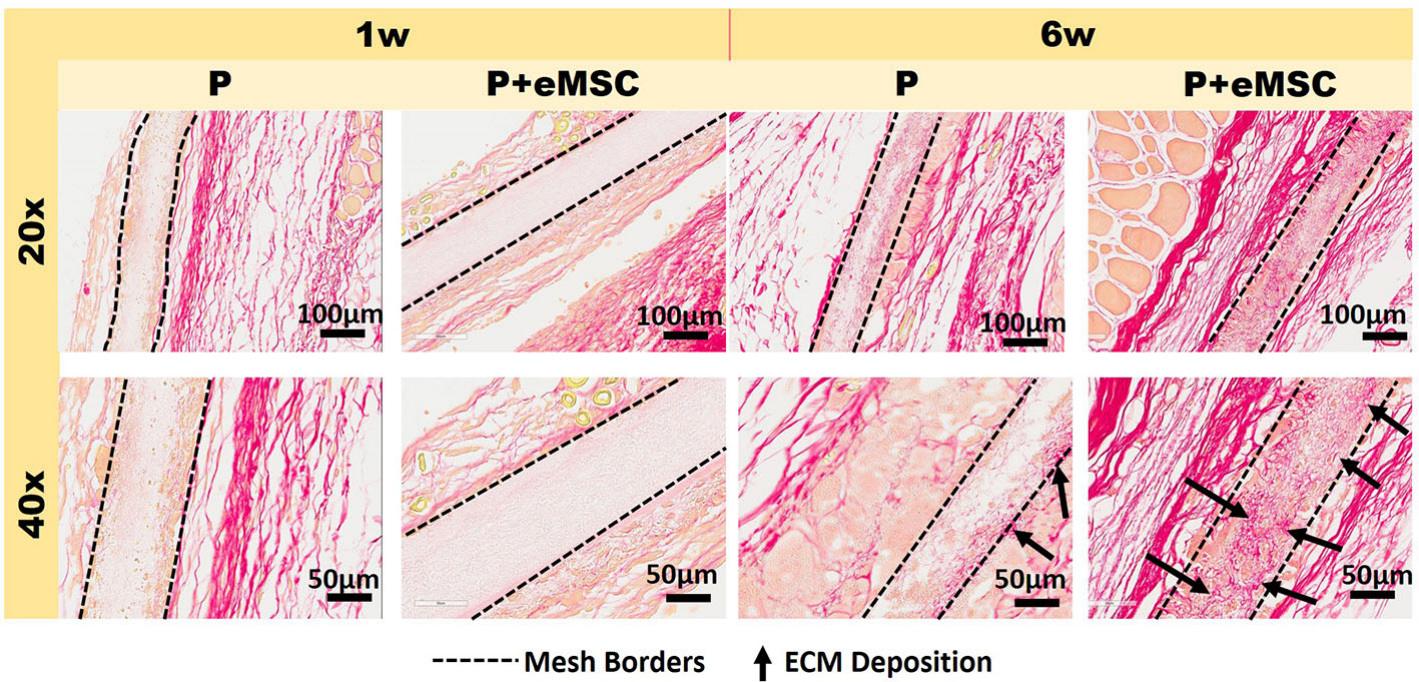
Picro-Sirius red staining of collagen in explanted meshes at 20× and 40× magnifications. P nanomesh implants (within dotted lines), with and without endometrial mesenchymal stem/stromal cells (eMSCs), explanted from the subcutaneous skin of the flank showing red-stained collagen red in mice at 1 and 6 weeks explantation. Black arrows show new collagen deposited within the P Nanomesh.

**Figure 3:**
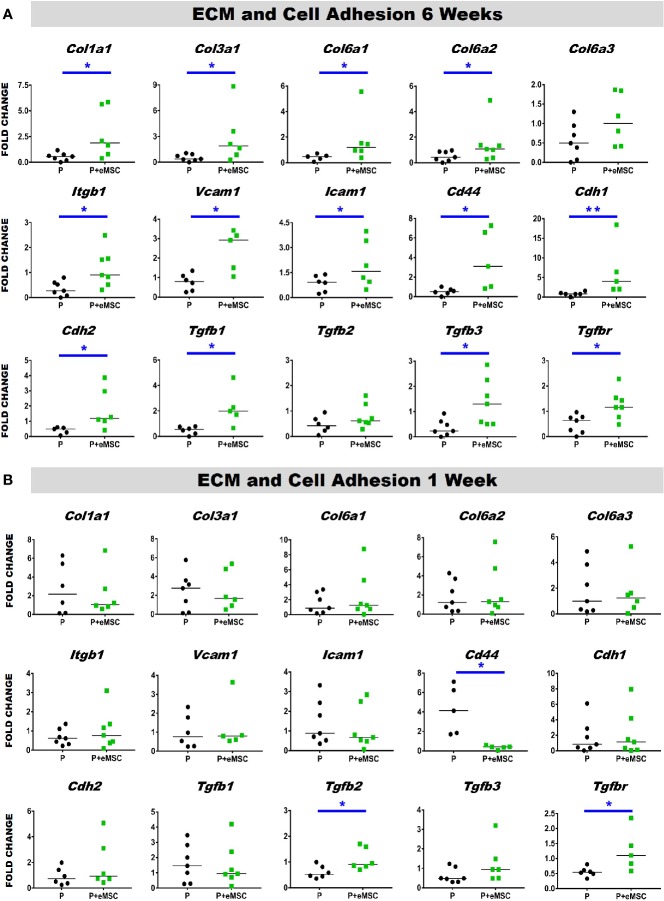
Extracellular matrix (ECM) and adhesion molecule gene expression of P and P+eMSCs nanomesh explants. Fold change in mRNA expression of ECM and cell adhesion genes by quantitative PCR in explanted mice tissues after **(A)** 6 weeks and **(B)** 1 week consisting of P nanomesh, with and without endometrial mesenchymal stem/stromal cells (eMSCs). Data are median of n=5-7 samples/group analyzed by Mann-Whitney U test; *P < 0.05 and **P < 0.01.

Histology sections prepared from mouse explants were stained with Picro Sirius red to visualize the newly synthesised ECM (**Figure 2**), mainly collagen (black arrows, **Figure 2**) inside implanted meshes (between dotted lines, **Figure 2**). At 6 weeks, there was a substantial amount of collagen inside the Nanomesh (within dotted lines, **Figure 2**) in the P+eMSC group compared to P alone. This difference was not observed at 1 week. Quantitative PCR analysis of ECM and genes (**Figure 3**) also showed a significant increase in the expression (P < 0.05) of several collagen genes including *Col1a1, Col3a1, Col6a1* and *Col6a2* in P+eMSC compared to P alone at 6 weeks (**Figure 3A**). Similar to histology observations (**Figure 2**), the expression of these genes was not different between the two groups at 1 week (**Figure 3B**). The expression of cell adhesion molecules and *Tgfb* genes, *Itgb1, Vcam, Icam, Cd44, Cdh1, Cdh2, Tgfb1, Tgfb3, and Tgfbr* were also significantly higher (P < 0.05) (**Figure 3A**), whereas expression of *Tgfb2* (**Figure 3A**) showed no difference between P+eMSC and P alone at 6 weeks. In contrast, at 1 week no collagen subunit genes were differentially expressed, neither for cell adhesion genes, except for *Cd44*, which was significantly lower in P+eMSC compared to P (**Figure 3B**). Our results show that presence of eMSC increases new collagen subunit synthesis which may be mediated by increased *Tgfb*1 and *Tgfb3* gene expression within implanted P nanomesh, may foster tissue integration *via* the expression of ECM formation and cell adhesion genes by 6 weeks but not as early as 1 week *in vivo*.

### eMSC Influence Expression of Matrix Metalloproteinases and Tissue Inhibitors of Metalloproteinases

**Figure 4:**
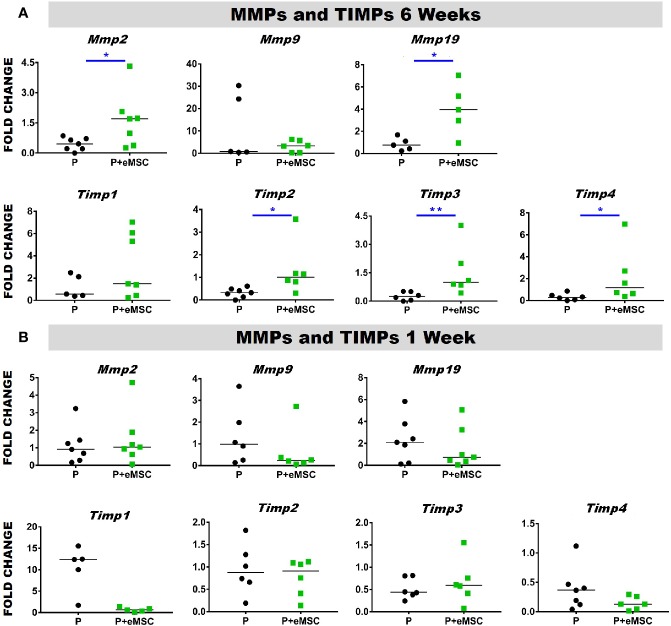
Matrix metalloproteinase (*Mmp*) and tissue inhibitors of metalloproteinases (*Timp*) gene expression of P and P+eMSCs nanomesh explants. Fold change in mRNA expression of mouse *Mmp* and *Timp* genes by quantitative PCR in explanted mice tissues after **(A)** 6 weeks and **(B)** 1 week consisting of P nanomesh, with and without endometrial mesenchymal stem/stromal cells (eMSCs). Data are median of n=5-7 samples/group analyzed by Mann-Whitney U test; *P < 0.05, **P < 0.01.

Matrix metalloproteinases (*Mmps*) are essential mediators of ECM homeostatic dynamics that degrade ECM components and are modulated by Tissue inhibitors of metalloproteinases (*Timps*). TIMPs reversibly bind to MMPs and regulate their proteolytic activities and their balance significantly impacts tissue homeostasis. To this end, we assessed the expression of several key *Mmp*s and *Timp*s using qPCR in *in vivo* tissues post implantation (**Figure 4**). At 6 weeks, the expression of *Mmp2, Mmp19*, *Timp2*, and *Timp3* was significantly higher (P < 0.05) in P+eMSC compared to P alone (**Figure 4A**). At 1 week, there was no significant differences in the expression of these MMPs and TIMPs genes in both groups (**Figure 4B**). Our results show that eMSC influence the expression of several MMPs and TIMPs when implanted as tissue engineered constructs compared with P nanomesh alone by 6 weeks.

### Nanomesh With eMSC Promote Angiogenesis After *In Vivo* Implantation

**Figure 5:**
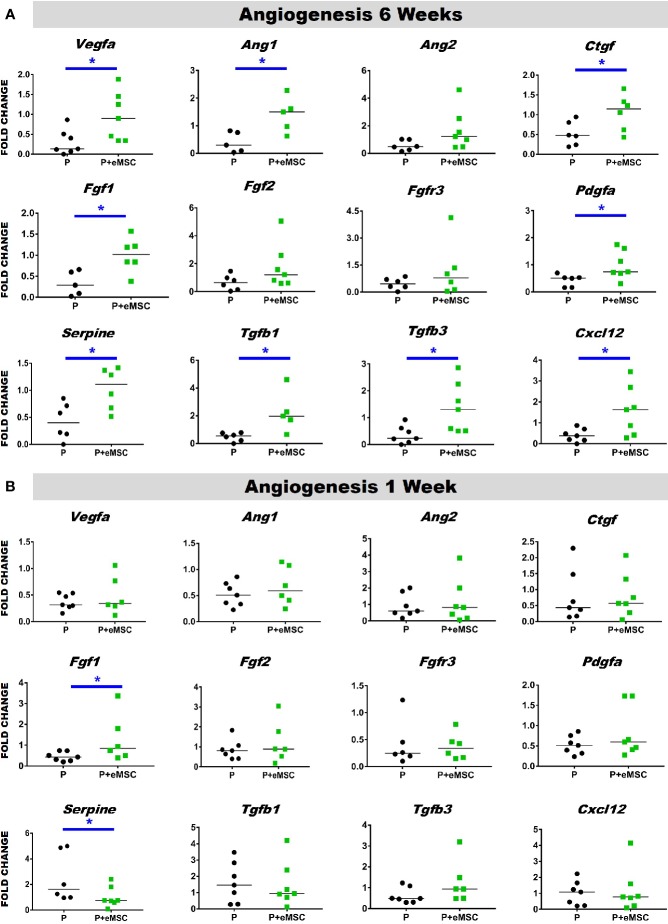
Angiogenesis gene expression of P and P+eMSCs nanomesh explants. Fold change in mRNA expression of mouse angiogenic genes by quantitative PCR in explanted mice tissues after **(A)** 6 weeks and **(B)** 1 week consisting of P nanomesh, with and without endometrial mesenchymal stem/stromal cells (eMSCs). Data are median of n=5-7 samples/group analyzed by Mann-Whitney U test; *P < 0.05.

**Figure 6:**
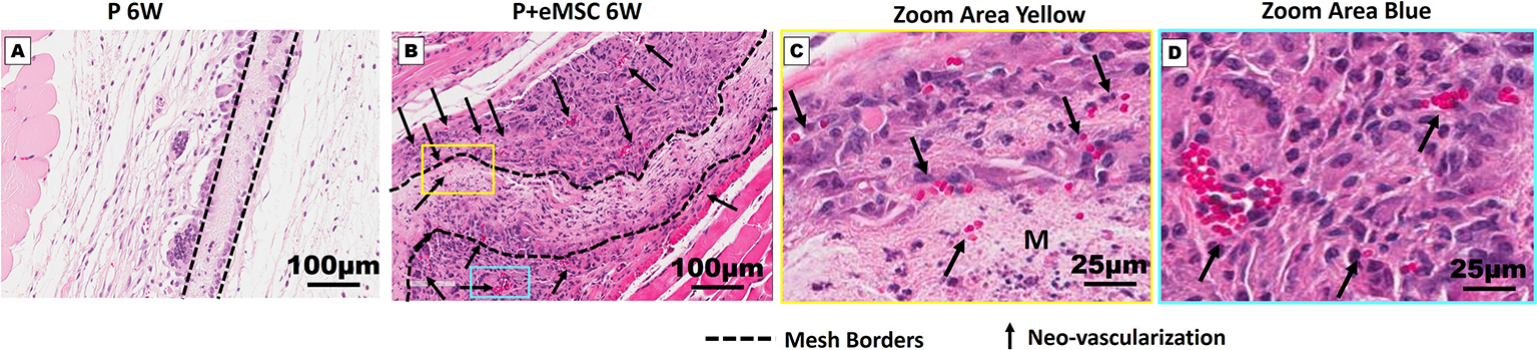
Neo-vascularization in Nanomesh explants after 6 weeks. H&E stained section showing mesh implants of **(A)** P and **(B)** P+eMSC at 6 week (dotted line). **(C)** Neo-vascularization (black arrows) around P+eMSC is seen at optical zoom within the yellow box area showing neo-vascular structures (black arrows) inside the mesh area (M) and **(D)** blue box area showing neo-vascular structures (black arrows) around the mesh area.

Angiogenesis is essential to healing and growth for repair of tissues. Rapid neo-vascularisation determines the clinical success of implanted tissue constructs. Since cells must be in 100–200 µm proximity of blood vessels to receive oxygen through diffusion, spontaneous ingrowth of capillaries is highly desirable following *in vivo* implantation of meshes (Shahabipour et al., 2019). Thus, it is critical that the post implantation milieu has optimal conditions to support vascularization for tissue integration and long-term viability. Therefore, we assessed the expression of several angiogenic factors following *in vivo* implantation of P nanomeshes with and without eMSCs at 1 and 6 weeks (**Figure 5**). At 6 weeks, we observed significantly higher expression (P < 0.05) of the key angiogenic factor genes, *Vegfa*, *Fgf1*, *Ctgf*, *Ang1*, and *Pdgfa* in P+eMSCs compared to P alone (**Figure 5A**). Of these, *Serpine* and *Fgf1* were also significantly higher in the presence of eMSC acutely at 1 week (**Figure 5B**), suggesting their role in a sustained angiogenic response. Expression of *Cxcl12*, a chemokine that plays a crucial role in angiogenesis by recruitment of endothelial progenitor cells (Li et al., 2015), was also significantly higher (P < 0.05) in the presence of eMSCs at 6 weeks (**Figure 5A**), however not at 1 week (**Figure 5B**), indicating involvement in late angiogenic responses. Our results indicate that, in comparison to P alone (**Figure 6A**), implantation of eMSC with P nanomesh promotes early angiogenesis and neovascularization as evidenced by H&E staining (**Figure 6B**) whereby several blood vessel profiles are located inside the mesh (black arrows, **Figure 6C**) as well as within a close proximity (10–200µm) of the mesh implant (black arrows, **Figure 6D**).

### eMSC Reduces the Pro-Inflammatory Response After Nanomesh Implantation

**Figure 7:**
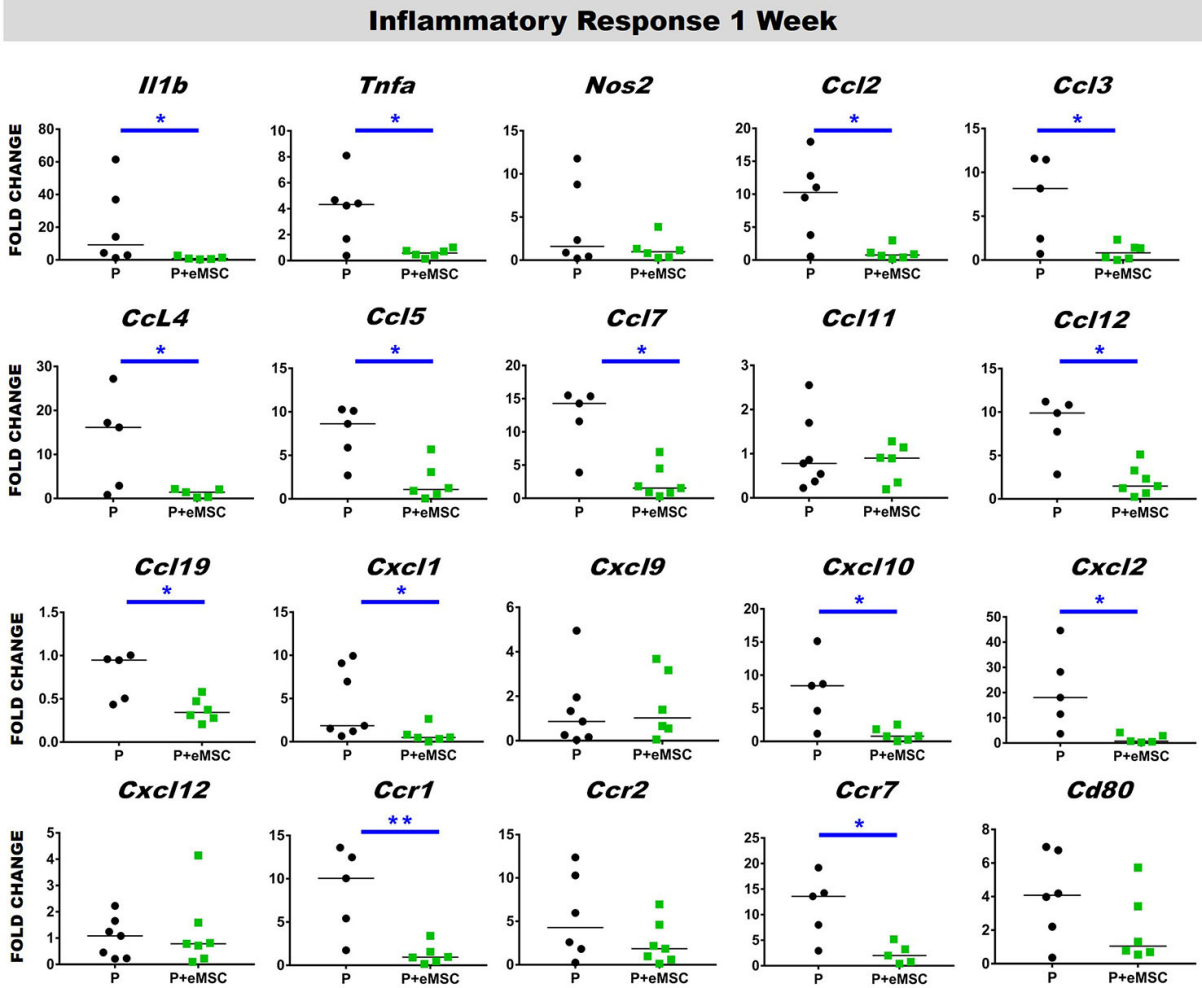
Acute Inflammatory gene expression after 1 week implantation of P and P+eMSC. Fold change in mRNA expression of mouse inflammatory genes by quantitative PCR in explanted mice tissues consisting of P nanomesh, with and without endometrial mesenchymal stem/stromal cells (eMSCs) after 1 week. Data are median of n=5-7 samples/group analyzed by Mann-Whitney U test; *P < 0.05 and **P < 0.01.

**Figure 8:**
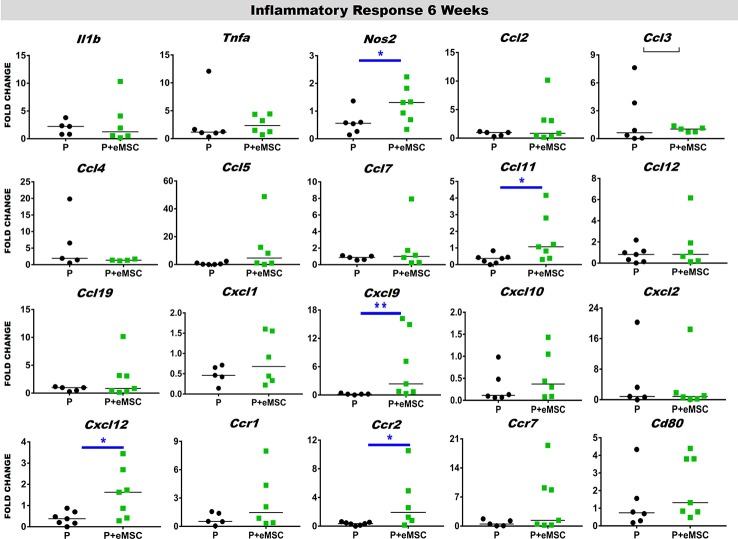
Inflammatory gene expression after 6 week implantation. Fold change in mRNA expression of mouse inflammatory genes by quantitative PCR in explanted mice tissues consisting of P nanomesh, with and without endometrial mesenchymal stem/stromal cells (eMSCs) after 6 weeks. Data are median of n=5-7 samples/group analyzed by Mann-Whitney U test; *P < 0.05 and **P < 0.01.

Immediately following mesh implantation, the immune system is triggered and an influx of white blood cells at the site marks the beginning of the FBR acute phase, which is characterized by several inflammatory cytokines. Several factors including components of the implants, determine the severity of this acute phase and the milieu of pro-inflammatory factors. Our analysis of pro-inflammatory factor genes showed that eMSC dampen and delay the expression of several acute pro-inflammatory genes in response to implanted P nanomesh (**Figures 7** and **8**). eMSC attenuated the inflammatory response associated with nanomesh at 1 week by significantly downregulating (P < 0.05) the expression of *Il1b*, *Tnfa*, *Ccl2*, *Ccl3*, *Ccl4*, *Ccl5*, *Ccl7*, *Ccl12*, *Ccl19*, *Cxcl1, Cxcl2, Cxcl10, Ccr1*, and *Ccr7* compared to P alone (**Figure 7**). In contrast to upregulating ECM and angiogenesis genes, we observed that all of these acute inflammatory genes were no longer upregulated at 6 weeks (**Figure 8**). However, the later phase inflammatory genes *Nos2*, *Ccl11*, *Ccxl9*, *Cxcl12*, and *Ccr2* which is the receptor for *Ccl11*, in P+eMSC were significantly upregulated (P < 0.05) compared to P alone. Our results show that eMSC seeded P nanomesh significantly and rapidly reduces the acute inflammatory response associated *in vivo* biomaterial implantation and therefore is likely to influence the entire subsequent FBR process. Some chemokines are upregulated in P+eMSCs at 6 weeks, associated with inflammatory cell recruitment functions such as *Cxcl12*.

### eMSCs Promotes an Anti-Inflammatory Response Following *In Vivo* Implantation at 6 Weeks

**Figure 9:**
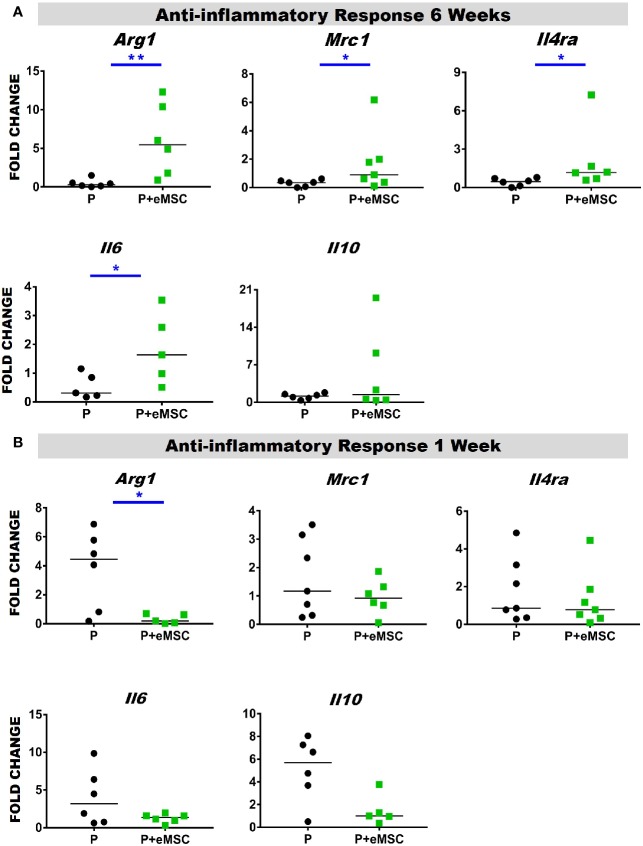
Anti-inflammatory gene expression of P and P+eMSCs nanomesh explants. Fold change in mRNA expression of mouse inflammatory genes by quantitative PCR in explanted mice tissues consisting of P nanomesh, with and without endometrial mesenchymal stem/stromal cells (eMSCs) after **(A)** 6 weeks and **(B)** 1 week. Data are median of n=5-7 samples/group analyzed by Mann-Whitney U test; *P < 0.05 and **P < 0.01.

Macrophages release of cytokines and growth factors induce migration and proliferation of fibroblasts, that in an anti-inflammatory environment effectively regenerate tissue (Koh and Dipietro, 2011). Accumulating evidence indicates that macrophages orchestrate the tissue response and healing process after biomaterial implantation and that macrophage polarization determines the outcome of the immune response (Ulrich et al., 2012; Roman Regueros et al., 2014; Feola et al., 2015; Darzi et al., 2016a). Herein, we observed a significantly higher (P < 0.05) expression of anti-inflammatory genes including *Arg1, Mrc1, Il6*, and *Il4ra* in P+eMSCs over P alone at 6 weeks (**Figure 9A**). While Il4ra is commonly associated with inflammation, recent evidence has shown it plays a role in M2 polarization by upregulating Il-6, another pro-inflammatory cytokine associated with tissue regeneration. Moreover, its ligand, Il-4 is commonly used to polarize M2 macrophages *in vitro* and is associated with wound repair. In contrast, *Arg1* was upregulated in the presence of P of eMSCs at 1 week (**Figure 9B**) which marks the acute phase of the FBR.

### Biomaterial-Induced Multinucleated Giant Cells With M2 Phenotype

**Figure 10:**
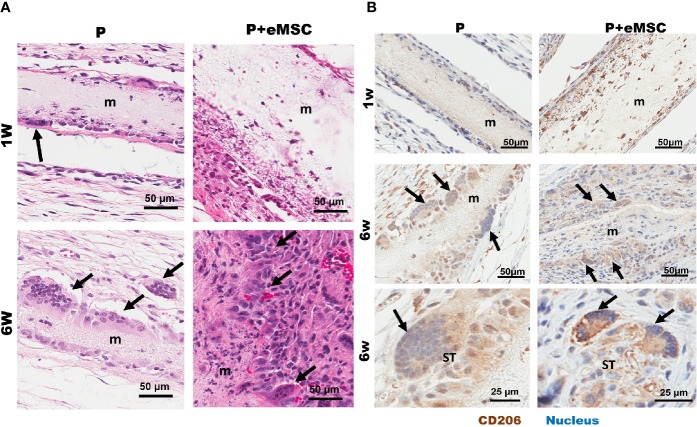
Characterization of Biomaterial-induced Multinucleated Foreign body Giant Cells (FBGC). **(A)** H&E staining of mice tissue sections comprising P nanomesh implants (m) with and without endometrial mesenchymal stem/stromal cell (eMSCs), in the subcutaneous flank between the abdominal wall and skin showing fusion of macrophages into multinucleated FBGC after 1 week and 6 weeks. **(B)** Multinucleated (blue stain) FBGCs (black arrows) show M2 type phenotype as characterized by CD206 (brown) immunostaining and are present along the mesh edges (m) and in the surrounding tissues (ST).

We found multinucleated foreign body giant cells (FBGC) associated with both P+eMSCs and P at 6 weeks but not at 1 week (**Figure 10**) by H&E stains, which showed fusion of macrophages mostly at the edges of the mesh (**Figure 10A**, black arrows). Our results show that the in the presence of eMSCs, the number of FBGCs were increased and smaller in size with fewer nuclei per FBGC. Immunohistological characterization revealed that these FBGCs expressed CD206, (**Figure 10B**, black arrows) a marker usually associated with M2 macrophages as shown within the mesh at 1 week in P+eMSC. These CD206 FBGCs were found both at the mesh edges and in the surrounding tissues. In P+eMSC, the intensity of CD206 in FBGC and other cells was greater, with respect to negative control (**Figure S2**) and localised to the plasma membrane compared to P alone at both time points. Although FBGC have been viewed in a negative light in FBR process, the knowledge of their role and functions remain elusive. Our study shows that these cells express CD206, a M2 marker and are present while there is a high expression of angiogenic, ECM synthesis, cell adhesion and anti-inflammatory genes in P+eMSCs at 6 weeks.

## Discussion

In this study, we report the difference in the profile of the FBR response, with focus on ECM, angiogenesis and inflammatory responses to degradable nanofiber meshes of PLCL (P) in the absence and presence of eMSCs. The main findings of our study are that MSCs promote better tissue integration of nanomesh through inducing increased expression of ECM, cell adhesion, angiogenesis and healing gene profiles 6 weeks following implantation, while dampening the pro-inflammatory response in the acute FBR phase at the first week. Given that the current failed vaginal meshes are associated with inadequate tissue integration and elevated chronic inflammatory FBR years after implantation (Nolfi et al., 2016), the use of eMSCs to reduce the pro-inflammatory response and promote early mesh integration and improve tissue repair is an important advance in improving outcomes for treating POP.

Our fabricated nanostructured porous P nanomeshes with a fiber diameter 585 nm (Mukherjee et al., 2019b) and a scaffold depth of ~406 nm have emerged as an attractive and potential alternative to nondegradable meshes owing to their biomimetic properties (Vashaghian et al., 2017; Gargett et al., 2019). Previously, we have shown that meshes with nano and micro architecture interact favorably with eMSC and promote their growth and proliferation (Mukherjee et al., 2019b; Paul et al., 2019). In the present study, our results show that the P nanomesh closely mimics the vaginal ECM architecture. Moreover, the bioengineering of nanomesh with eMSCs triggers a distinctly more favorable immune and tissue response *in vivo* compared to nanomesh alone. From a clinical perspective, in targeting POP treatment, these are highly desirable features as nanostructured meshes recapitulate structural cues for cell adhesion and prolonged retention of large numbers of MSCs after local delivery even after 6 weeks (**Figure S3**).

Following implantation of a biomaterial construct *in vivo*, a macrophage-mediated FBR is triggered whereby several molecular mechanisms are activated at each step of the process (Anderson et al., 2008; Skokos et al., 2011; Mooney et al., 2014; Mukherjee et al., 2019a). Accumulating evidence from our and other groups indicate that macrophages orchestrate the tissue response and healing process after biomaterial implantation and that macrophage polarization determines the outcome of the immune response (Ulrich et al., 2012; Roman Regueros et al., 2014; Feola et al., 2015; Darzi et al., 2016a; Paul et al., 2019). Our results show that eMSC-based nanomesh implants corroborate known M2 macrophage effects after 6 weeks implantation in mice by increased expression of *Il10 and Tgfb* genes which induced the M2 phenotypic genes *Arg1* and *Mrc1* on the accumulating macrophages. eMSCs also induced upregulation of angiogenic genes *Tgfbr, Vegfa, Ang1*, and *Pdgfa* promoting neovascularisation around the meshes and the chemotactic and recruitment genes *Cxcl12, Ccr2*, and *Ccl11* to promote the initial macrophage accumulation around the implanted mesh. Therefore, it is clear that eMSCs have a direct impact on the host macrophages by polarizing them to an M2 phenotype and proactively modulating their response to the implanted biomaterial *in vivo* that promotes nanomesh integration.

Once macrophages are recruited to the implant surface, they begin to accumulate and release chemo-attractive signals such as TNF-α, IL-1β, IL-6, and CCL2 that further enhances macrophage assembly at the interface (Anderson et al., 2008; Van Linthout et al., 2014; Kyriakides, 2015). Our results show that a number of chemotactic genes (*Ccr2, Ccl11, Cxcl9*, and *Cxcl12*) are upregulated in the eMSC-containing constructs and their reduction over time correlates with the progression of the FBR in a modulated manner at the surface tissue interface. This is evident from significantly elevated expression of cell adhesion genes *Itgb1*, *Cdh1*, *Cdh2*, *Vcam1*, and *Cd44*. The JAK/STAT signalling pathway is activated in the FBR when IL-4 binds to its receptor on macrophages, inducing the phosphorylation of STAT6 which translocates to the nucleus and upregulates the expression of E- cadherin or N-cadherin and β-catenin (Moreno et al., 2007). Upregulation of this adhesion molecule enhances cell-cell interactions, induces the fusion of macrophages (Mcnally et al., 1996; Van Linthout et al., 2014) and modulates the M2 response, mainly *in vitro* (Van Den Bossche et al., 2015). After macrophages are bound *via* their integrin receptors, downstream signal transduction can affect cytoskeletal rearrangement and formation of more adhesion structures allowing macrophages to spread over the biomaterial surface as we observed in this study. This spreading is facilitated by specialized macrophage podosomes consist of actin filaments that are associated with both initial macrophage adhesion and subsequent macrophage fusion to form FBGCs (Kyriakides, 2015; Chung et al., 2017). Our results indicate that eMSCs modulated several cell adhesion genes to promote a coordinated interaction with the biomaterial and promote graft tissue integration, albeit at a later time point. Such a response is critical to favorable long-term outcomes after mesh implant-based POP reconstructive surgery.

Nanomeshes themselves may not have the mechanical properties to alleviate POP symptoms and therefore, we envision they augment native tissue repair surgery. However, over time, the bioengineered nanomeshes can stimulate the body to produce ECM which will not only drive tissue integration but also provide sufficient mechanical strength to the vaginal wall to prevent further herniation of pelvic organs into the vagina in POP following surgery. Herein, we observe that eMSC promoted a synergistic action between expression of matrix formation genes such as *Col1a1*, *Col3a1*, *Col6a1*, *Col6a2*, and other genes associated with fibroblast activity such as *Tgfb* as well as the ECM regulation genes, *Mmps* and *Timps* after 6 weeks of nanomesh implantation. In general, fibroblasts are the cells responsible for maintaining ECM homeostasis (Kastellorizios et al., 2015) by producing and remodelling ECM, mediated by tightly regulated and opposing activities of *Mmps* and *Timps*. Given the balanced expression of ECM forming and regulating genes, eMSCs promote recruitment of fibroblasts (**Figure S4**) to maintain this homeostatic balance in the tissue environment. Fibroblasts are chemotactically attracted to the site of injury such as *Cxcl12*, where they are induced to proliferate and secrete ECM in a process referred to as fibroplasia (Kendall and Feghali-Bostwick, 2014). Indeed, several ECM genes are upregulated in presence of eMSCs. Therefore, it is likely that fibroblasts participate in the later stages of inflammation by responding to Tgfb1, Il1b and Il6, proteins which increase matrix production, in addition to lipids such as prostaglandins and leukotrienes (Kendall and Feghali-Bostwick, 2014; Jones, 2015). Fibroblasts can also produce TGFβ1, IL-1β, IL-33, CXC and CC-chemokines, and ROS, which serve to recruit and activate macrophages (Kendall and Feghali-Bostwick, 2014; Jones, 2015). Our results indicate that eMSC influence gene expression of these factors released in repairing tissues such as *Tgfbs*, *Il1b*, and *Il6*. Several CXC and CC-chemokines impacting cellular recruitment and angiogenesis such as *Ccl2*, *Ccl3*, *Ccl4*, *Ccl5*, *Ccl12*, *Cxcl2*, *Cxcl3*, and *Cxcl10* were also upregulated by the presence of eMSCs highlights their influence on host fibroblast activity. MMP and TIMP are also produced by macrophage themselves that influence remodelling in the local environment.(Nakashima et al., 1998; Laquerriere et al., 2004). Macrophages themselves are known to release MMPs such as MMP1, 2, and 9 in proportions related to biomaterial debris around the bone prosthetic materials. Nonphagocytable particles showed more MMP-9 where as phagocytable debris were associated with larger amounts of Il-1β.(Laquerriere et al., 2004) Although most of these studies are performed around bone remodelling, they show that macrophage response to biomaterials may be driven by local environment conditions. While the formation of ECM is often associated with fibrosis and its deleterious effects of encapsulating mesh, we show a higher and balanced expression of MMPs and TIMPs together with increased *Tgfb*, a fibroblast stimulator. This finding indicates that ECM formation is highly regulated and that the presence of eMSC controls and minimizes fibrosis at 6 weeks and in turn stimulates *Tgfb1* expression. A balance between TGFβ and TIMP-1also aids nonfibrotic tissue repair (Jones, 2015). *Tgfb* also causes fibroblast deposition of ECM and secretion of many paracrine and autocrine growth factors, including CTGF (Leask et al., 2009) as confirmed by increased *Ctgf* gene expression in the presence of eMSCs. In humans, CTGF is involved in angiogenesis, cell migration, adhesion, proliferation, tissue wound repair, and ECM regulation and is induced by TGFβ and IL-1β (Kendall and Feghali-Bostwick, 2014; Jones, 2015). CTGF also binds to ECM proteins and growth factors including VEGF and TGFβ to maximally induce type I collagen synthesis, α-SMA and also increases *IL6* expression (Jun and Lau, 2011; Liu et al., 2012). Our gene expression results along with co-localization studies (**Figure S4**) suggest that the paracrine effects of eMSCs directly influence these molecular pathways to regulate, synthesise and maintain ECM, even within nanomeshes as seen in **Figure 2**.

Inflammatory factors and adhesion molecules such as ICAM and VCAM recruit monocytes, mast cells, and fibroblasts, all of which can produce proangiogenic factors, including VEGF and FGF through a cascade of cell and chemokine interactions (Mahdavian Delavary et al., 2011). Our results showed that eMSC increased *Vcam*, *Cxcl12*, and *Ccr2* suggesting that these in turn increased expression of the proangiogenic factors *Vegfa*, *Fgf1*, and *Ang1* to promote angiogenesis. VEGF-A directly stimulates endothelial cell proliferation by engaging with the VEGFR-2 to activate its tyrosine kinase domain and initiate the sprouting of new vessels from existing micro-vessels (Mahdavian Delavary et al., 2011). VEGF, likely produced by the recruited macrophages and fibroblasts, may also contribute to the angiogenic process by mobilizing endothelial progenitor cells and other myeloid cells to the site of angiogenesis (Antonella and Fabio, 2005). Overall, the neovascularisation of the nascent ECM is critical for ensuring viability of the new tissue surrounding the nanomesh. The upregulation of proangiogenic chemokine genes such as *Ccl11* and its receptor Ccr2, *Cxcl9*, and *Cxcl10* to some extent at 6 weeks after P implantation, suggest these are key players in initiating angiogenesis and fibroblast activity. In humans, CCL11 and well as CXCL10 are known to recruit eosinophils that subsequently induce a prolonged angiogenic effect (Van Linthout et al., 2014). This signifies that eMSC promote angiogenesis through paracrine effects even after mesh implantation. Our results also show eMSC increase *Pdgfr*, *Ang1*, and *Tgfb* expression, that are associated with pericytes to stabilize nascent endothelial cell tubes during angiogenesis (Antonella and Fabio, 2005). These results are highly encouraging given that blood vessels were found in close proximity to the P nanomesh, a highly desirable tissue engineering outcome (Shahabipour et al., 2019), and completely integrated with tissue by 6 weeks suggesting a highly influential role for eMSC in modulating the entire FBR process.

Our previous studies have shown that bioengineering of eMSCs indeed modulates the FBR process to both nondegradable (Ulrich et al., 2014; Darzi et al., 2018; Emmerson et al., 2019) and degradable nanomeshes (Mukherjee et al., 2019b; Paul et al., 2019) and can be detected *in vivo* even upto 6 weeks after implantation (**Figure S3**). Yet, we showed that eMSC facilitated M2 polarization of macrophages with immune-regulatory properties that dampen inflammation. In this cascade upregulation of cytokine and chemokine genes such as *Ccl7*, that also indirectly influence the adaptive Th2 immune system as they recruit other innate immune cells such as basophils and mast cells (Biswas and Mantovani, 2010). They also promote angiogenesis and wound healing *via* the production of PDGFA and VEGF (Antonella and Fabio, 2005; Martinez et al., 2006; Martinez et al., 2008) genes upregulated by the presence of eMSCs. Macrophages participate in a number of ways to regenerate tissue and heal wounds through a cascade of inflammatory responses, thereby contributing to tissue ECM formation. eMSCs promote *Mmp19 and Mmp2* that degrade the ECM (Jones, 2015; Mukherjee et al., 2019a) which releases growth factors and chemokines (Detry et al., 2012) respectively. The upregulation of MMP2 and MMP19, which are promoters of angiogenesis through release of ECM growth factors (Webb et al., 2017; Liu et al., 2018b) likely contributed to the angiogenesis observed in our study. While we note the increase in *IL-6* and *IL-4ra*, which are mostly associated with pro-inflammatory responses, they also have anti-inflammatory roles (Fuster and Walsh, 2014). A recent landmark study has shown that IL-6 primes macrophages for IL-4-dependent M2 polarization by inducing IL-4RA expression *via* STAT3-mediated activation of the IL4ra (Mauer et al., 2014). Thus, macrophages have different functions during the healing, macrophage process (Galdiero and Mantovani, 2015; Rőszer, 2015). This is clearly seen in our results, where eMSCs increased the expression of genes associated with the M2 macrophage phenotype and the healing response.

The presence of FBGCs after biomaterial implantation is often viewed as a negative response and has been directly linked to FBR leading to material rejection. Recent accumulating evidence questioned the role of FBRCs in these deleterious effects. Both *in vitro* and *in vivo* studies have shown that FBGC exhibit different phenotypic profiles, in particular the expression of both pro and anti-inflammatory cytokines, depending on the physicochemical characteristics of the biomaterials (Ghanaati et al., 2010; Mcnally and Anderson, 2015). Herein, we showed FBGCs with an M2 phenotype with differences in their fusion pattern based on the cellular component (ie eMSC) of the bioengineered implant. Recent reports have indicated that FBGCs are a potent source of VEGF, promote mannose receptor mediated phagocytic processes and may be involved in the process of implant bed vascularization by stimulating angiogenesis (Mcnally and Anderson, 2011; Mcnally and Anderson, 2015; Barbeck et al., 2016). In agreement, we showed CD206 expressing FBGC in P+eMSC explants after 6 weeks, together with significant upregulation of several angiogenic genes and formation of neo-vessels. Moreover, several chemokines and cell adhesion genes which were down regulated at 1 week but upregulated at 6 weeks, may be involved in the recruitment and fusion of macrophages to form FBGC. MSC incorporated biomaterials modulate bone healing by formation of FBGC that ultimately lead to angiogenesis and long term stability of implants in humans.(Miron and Bosshardt, 2018) Given their capacity to promote both tissue inflammatory and/or tissue wound healing, the appropriate characterization of FBGCs is therefore critical. While, further studies are pivotal to establish their exact role and mechanisms of cell–cell communication, our study suggests that they may be closely associated with rapid establishment of homeostasis after implantation by aiding in key tissue repair processes.

Since the discovery of eMSCs, it has been applied to various areas of research including POP treatment (Gargett et al., 2016). Our research has shown that eMSCs can modulate FBR to various types of meshes, a phenomenon we suspected to be a paracrine effect(Gargett et al., 2019). Moreover, there is an urgent unmet health need and heavy drive in design of biomaterials that can be used for regenerative medicine, including POP treatment (Mukherjee et al., 2010; Gargett et al., 2019). These approaches include surface modifications and growth factor release from materials to modulate the FBR and repair process. This study provides an insight into the gene expression profile of host response that are modulated by eMSCs that is likely to aid researchers in the field of biomaterials and regenerative medicine with evidence and knowledge to better design constructs. Our results also help to understand FBR processes that are particularly impacted by eMSCs and will enable future studies in uncovering the exact mechanisms to hopefully overcome the current hurdles in clinical care.

## Conclusion

In summary, our study provides the first extensive profiling of gene expression following P nanomesh implantation and the impact of tissue engineering them with eMSCs. Our results show that eMSC, most likely through their paracrine effects, significantly modulate the elicited FBR. In particular, eMSCs induce upregulation of ECM, cell adhesion and angiogenic genes, most likely through the increased expression of several chemokines and cytokines at 6 weeks but not acutely at 1week. However, in the absence of eMSCs, the acute response is pro-inflammatory, while the presence of eMSCs leads to a M2 healing response after 6 weeks following P nanomesh implantation. Thus, the initial alterations to the FBR mediated by eMSCs show longer term favorable outcomes. The expression of these genes collectively leads to the formation newly synthesized ECM within the nanomeshes and neo-vasculature in close proximity. From a tissue engineering perspective, this is a hallmark of a highly successful implant and will likely overcome the current hurdles faced in POP treatment.

## Study Limitation

(1) This study used tissues that were close to the meshes implanted for the gene expression study. Although ideal, it was not feasible to extract the cells that infiltrated the mesh due to technical challenges. (2) This study was performed in a mice subcutaneous model rather than vaginal model owing to the small size of mouse vagina. Further studies in larger animal models are needed to fully understand the exact immunogenic properties of these constructs in the vaginal environment.

## Data Availability Statement

The data generated for this study can be found in GEO using accession number GSE141960.

## Ethics Statement

The studies involving human participants were reviewed and approved by Monash Health and Monash University Human Research Ethics committees (09270B). The patients/participants provided their written informed consent to participate in this study. The animal study was reviewed and approved by Monash Medical Centre Animal Ethics Committee A (2017/05).

## Author Contributions

Study conception and design: SM, SD, JW, CG. Ethics and animal care: SM, SD, FC, KP, CG. Experiment design: SM, SD, FC. Perform experiment: SM, SD, KP. Statistical analysis: SM, SD, FC. Manuscript writing and editing: SM, SD, CG, JW.

## Funding

This work was financially supported by National Health and Medical Research Council (NHMRC) of Australia Project Grant (Grant No. 1081944 and 1159677) and Senior Research Fellowship (Grant No. 1042298 for CEG); Science and Industry Endowment Fund (John Stocker Fellowship Grant no. PF16-122 for SM); Rebecca L Cooper Medical Research Foundation (Grant no. 10770), Evans Foundation (formerly Youanmi Foundation); CSIRO, Clayton Australia and the Victorian Government's Operational Infrastructure Support Program.

## Conflict of Interest

The authors declare that the research was conducted in the absence of any commercial or financial relationships that could be construed as a potential conflict of interest.

The handling editor declared a shared affiliation, though no other collaboration, with the authors SM, SD, FP, FC, JW, CG at time of review.
