# Supplementary material for: Electrospun Nanofiber Meshes With Endometrial MSCs Modulate Foreign Body Response by Increased Angiogenesis, Matrix Synthesis, and Anti-Inflammatory Gene Expression in Mice: Implication in Pelvic Floor
Source: Front Pharmacol. 2020 Mar 24;11:353. doi: 10.3389/fphar.2020.00353 (PMC7107042; doi:10.3389/fphar.2020.00353)
Supplement: Supplementary file 1 [file DataSheet_1.pdf]

SUPPLEMENTARY DATA

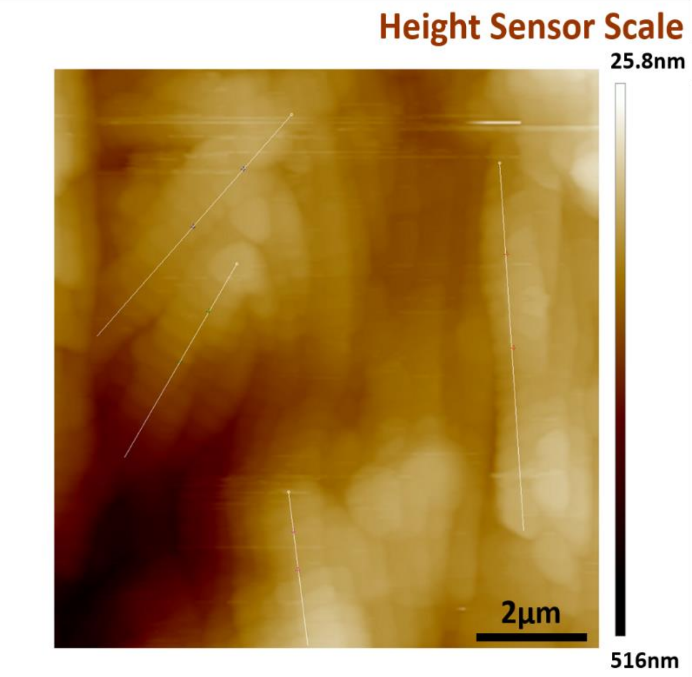

Figure S1: Atomic force Microscopy of human vaginal tissue showing height sensor scale corresponding to Fig 1E.

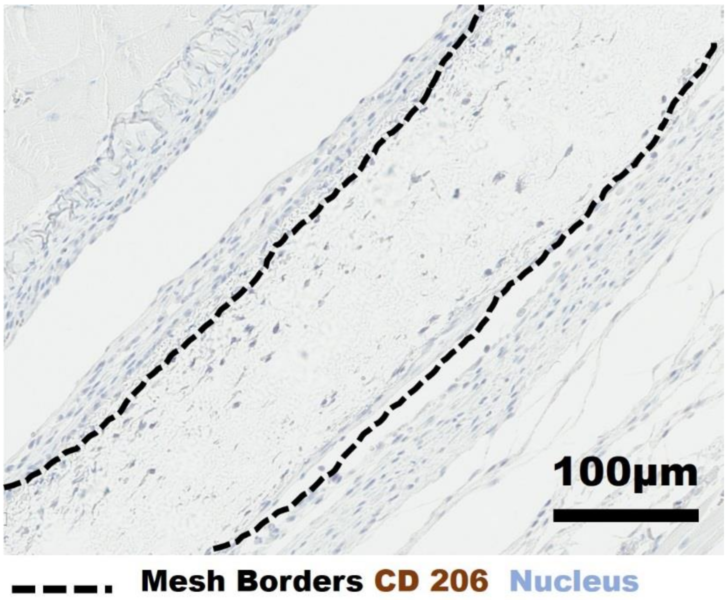

Figure S2: Negative control for immunohistochemical staining of CD206 of mice tissue with nanomesh implant

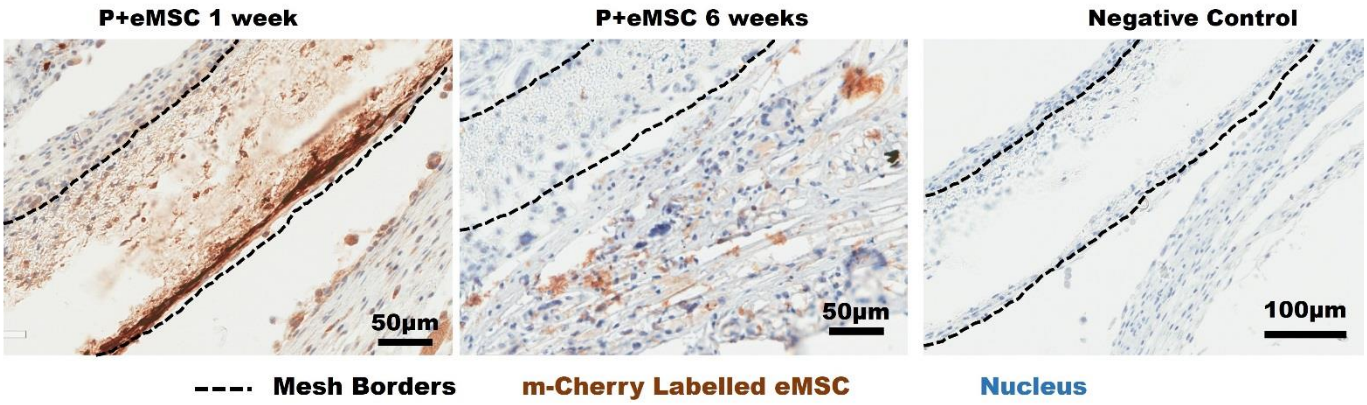

Figure S3: Immunohistochemically stained m-Cherry lentivirus labelled eMSCs in mice tissue with P nanomesh implant using anti m-Cherry antibody after 1 week and 6 weeks compared to negative control.

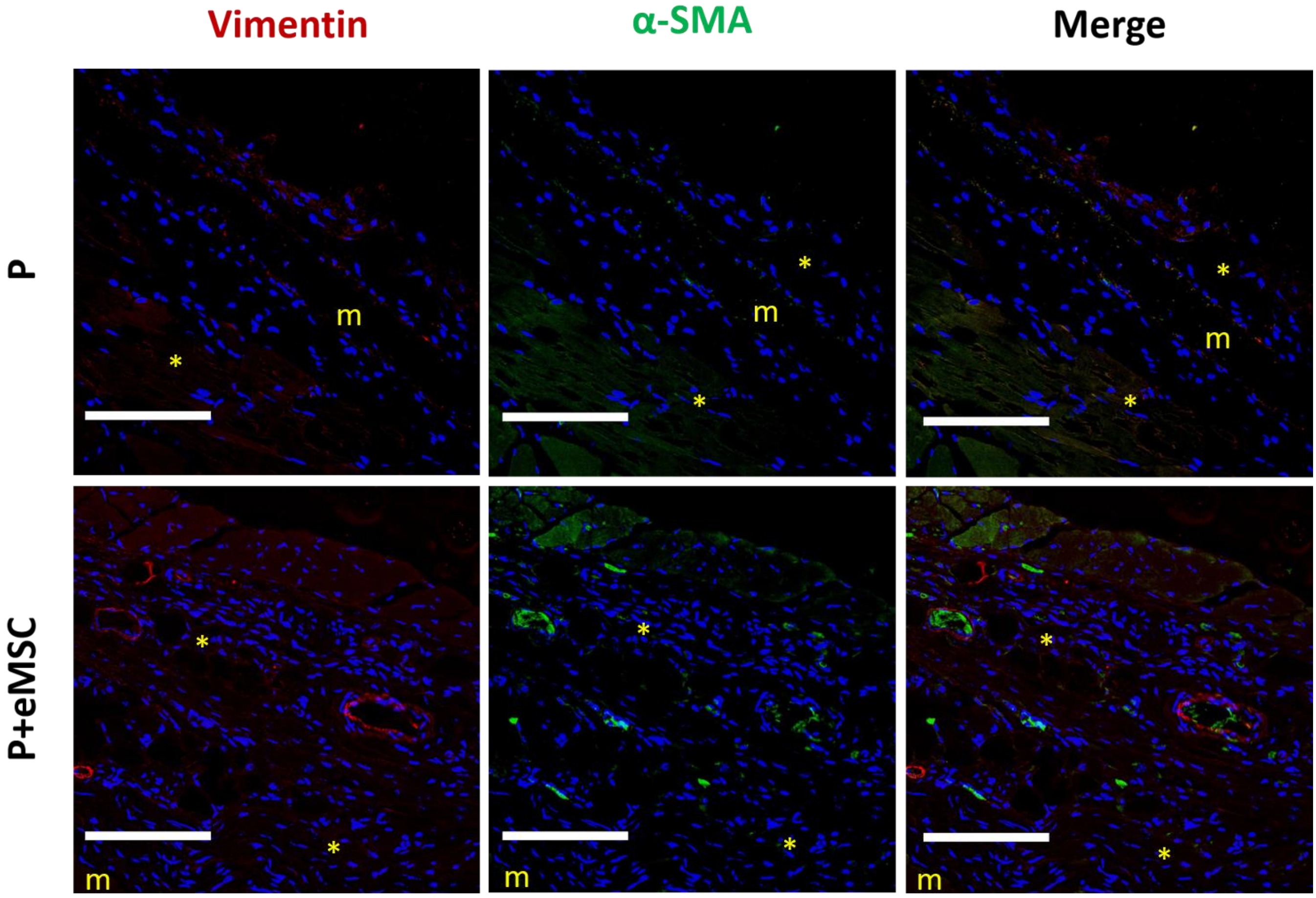

Figure S4: Immunofluorescence to characterize cells in mice tissues (*) around nanomesh implants (m) after 6 weeks using vimentin and alpha smooth muscle actin (scale bar= 100µm).
